# Associations Between Urinary Metabolic Indicators and Metabolic Dysfunction–Associated Steatotic Liver Disease and Liver Fibrosis: Evidence from National Health and Nutrition Examination Survey 2017-2020

**DOI:** 10.5152/tjg.2026.26228

**Published:** 2026-09-08

**Authors:** Jiacheng Cai, Hong Wang, Leilei Wang, Lei Zhang

**Affiliations:** The Third People’s Hospital of Yinzhou District, Zhejiang, People’s Republic of China

**Keywords:** CAP, LSM, MASLD, NHANES, liver fibrosis, urinary metabolic indicators

## Abstract

**Background/Aims:**

Metabolic dysfunction–associated steatotic liver disease (MASLD) reflects the central role of systemic metabolic disturbances in the pathogenesis of fatty liver. Urinary metabolic indicators may capture integrated renal, inflammatory, and metabolic alterations associated with MASLD and liver fibrosis.

**Materials and Methods:**

The authors analyzed 3496 adults from the 2017-2020 National Health and Nutrition Examination Survey cycles. Urinary metabolic indicators—including serum uric acid-to-creatinine ratio (SUACR), urinary albumin-to-creatinine ratio (UACR), uric acid-to-high-density lipoprotein cholesterol ratio (UHR), uric acid-to-albumin ratio (UAR), and blood urea nitrogen-to-creatinine ratio (BUN/Cr)—were assessed. MASLD and liver fibrosis were defined using controlled attenuation parameter (CAP) and liver stiffness measurement (LSM), respectively. Associations were evaluated using multivariable regression models adjusted for demographic, lifestyle, and cardiometabolic factors.

**Results:**

Higher SUACR, UACR, UHR, and UAR were positively associated with CAP and MASLD (all *P* < .05), whereas BUN/Cr was not significantly associated with either outcome. For liver fibrosis, SUACR, UHR, and UAR remained independently associated after multivariable adjustment. Nonlinear, threshold-dependent patterns were observed for SUACR, UHR, and UAR with MASLD and for UHR with fibrosis. Among the urinary metabolic indicators, UHR demonstrated the strongest predictive performance for MASLD, whereas UACR demonstrated the best predictive performance for fibrosis.

**Conclusion:**

Urinary metabolic indicators, particularly uric acid–related indicators, were associated with MASLD and liver fibrosis, suggesting that they may reflect systemic metabolic and renal dysregulation. These findings support their potential utility for noninvasive risk assessment and warrant further investigation into their mechanistic role in the progression of fatty liver disease.

**Main Points**

- Urinary metabolic indicators were evaluated in relation to hepatic steatosis and fibrosis, using controlled attenuation parameter and liver stiffness measurement as surrogate measures of steatosis and fibrosis.
- Urinary metabolic indicators exhibited stable positive associations with metabolic dysfunction–associated steatotic liver disease and elevated liver stiffness, supporting their utility as markers of systemic metabolic and renal dysregulation.
- This study provides new epidemiological evidence of associations between composite urinary metabolic indicators and liver fibrosis, an area that remains relatively understudied.
- Nonlinear and threshold-dependent associations were observed for several indicators, suggesting that their relationships with MASLD and liver fibrosis may vary across exposure levels and may offer additional value for refined risk stratification.

## Introduction

Hepatic steatosis in conjunction with cardiometabolic dysfunction—such as obesity, type 2 diabetes mellitus (T2DM), or dyslipidemia—is now classified under the recently proposed term metabolic dysfunction–associated steatotic liver disease (MASLD), which replaces the traditional concept of nonalcoholic fatty liver disease (NAFLD).^1^ This change reflects a shift in the conceptual framework toward the recognition of underlying metabolic drivers. MASLD is currently the predominant form of chronic liver disease worldwide, affecting approximately 25% of adults.^2^ By prioritizing metabolic dysfunction in its definition, MASLD represents a conceptual shift from NAFLD, which was largely defined by the exclusion of alcohol consumption and other liver diseases.^2^ This updated framework offers a more mechanistic classification and adopts an inclusion-based diagnostic approach, enhancing its clinical relevance to cardiometabolic risk while reducing the potential for stigmatizing terminology.^3^ The inclusion of subtypes such as metabolic and alcohol-related liver disease further supports a more nuanced classification of patients with combined metabolic and alcohol-related liver disease.^4^ However, limitations remain, including potential heterogeneity arising from broader diagnostic criteria and challenges in transitioning established epidemiological data and clinical practices from NAFLD to MASLD.^5^ Overall, although MASLD largely overlaps with NAFLD in clinical populations, its updated definition provides a more pathophysiologically grounded and clinically actionable framework for both research and patient management.

In recent years, several renal and metabolic composite indices derived from routine laboratory measurements have attracted increasing attention because of their potential to capture systemic metabolic, inflammatory, and microvascular alterations. The serum uric acid-to-creatinine ratio (SUACR) integrates endogenous purine metabolism with renal excretory function, potentially providing a more stable indicator than uric acid (UA) alone and demonstrating utility in cardiometabolic and renal risk stratification.^6^ The urinary albumin-to-creatinine ratio (UACR), derived from urinary albumin and creatinine measurements, is widely used to detect early renal dysfunction and microalbuminuria in large-scale studies.^7^ Blood urea nitrogen-to-creatinine ratio (BUN/Cr), another widely used index, reflects the dynamic balance between protein metabolism and kidney filtration and provides insight into both renal perfusion and systemic metabolic state.^8^ More recently, integrative biomarkers such as UA to high-density lipoprotein cholesterol (HDL-C) ratio (UHR) have been introduced to simultaneously reflect metabolic and inflammatory alterations, with higher UHR values associated with a less favorable cardiometabolic profile.^9^ Furthermore, the uric acid-to-albumin ratio (UAR) has recently emerged as a potential integrative marker linking oxidative stress, nutritional status, and systemic inflammation, although its clinical applicability remains to be established.^10^ Collectively, these indicators reflect not only renal function but also broader metabolic dysregulation, suggesting their potential relevance to systemic metabolic diseases such as MASLD.

Hepatic steatosis and fibrosis can be quantitatively evaluated using vibration-controlled transient elastography (VCTE), a validated noninvasive method that provides controlled attenuation parameter (CAP) and liver stiffness measurement (LSM) values, which reflect hepatic steatosis and fibrosis, respectively.^11^ Although prior studies have examined the associations between selected urinary metabolic indicators and MASLD,^12^^,^^13^ their potential relationships with hepatic fibrosis remain incompletely characterized. Moreover, much of the existing evidence has relied on indirect or surrogate measures of hepatic steatosis, such as the fatty liver index and the United States fatty liver index rather than VCTE-based measurements. Consequently, the associations of these indicators with MASLD defined using both CAP and LSM have not been systematically evaluated. Importantly, several emerging composite biomarkers, particularly those incorporating UA-related metrics, remain largely underexplored in relation to both MASLD and hepatic fibrosis. Accordingly, leveraging VCTE-based phenotyping in large, population-based datasets provides a more robust and clinically relevant framework for elucidating these associations and addressing critical gaps in the current literature. Using data from National Health and Nutrition Examination Survey (NHANES), the authors evaluated the cross-sectional associations between urinary metabolic indicators and hepatic outcomes aimed to better characterize the metabolic and inflammatory processes underlying MASLD and liver fibrosis.

## Materials and Methods

### Study Population

This study used data from NHANES 2017-2020, a publicly available dataset containing deidentified participant information; therefore additional ethical approval was not required. Biochemical markers, including fasting glucose, lipid parameters, and liver function indices, were measured according to standardized NHANES protocols in certified laboratories using automated analyzers. Because the data were deidentified, additional informed consent was not required. Of the 15 560 participants initially identified, those without liver elastography data, with hepatitis B or C infection, or with excessive alcohol consumption were excluded. Participants with incomplete key covariate data (n = 2633) or incomplete laboratory data required for calculating urinary metabolic indices (n = 1007) were also excluded. The final analytic cohort comprised 3496 participants (Figure 1).

### Biochemical Measurements and Calculation of Urinary Metabolic Indicators

Biochemical and urinary analytes in NHANES were measured using standardized laboratory protocols. Urine samples were collected at the mobile examination center, and urinary albumin and creatinine concentrations were measured using fluorescent immunoassay and enzymatic techniques to calculate the UACR (mg/g). Fasting serum concentrations of UA, BUN, Cr, HDL-C, and albumin were measured in certified laboratories using automated assays with standardized quality control procedures. These variables were combined to derive composite indices, including SUACR, BUN/Cr, UHR, and UAR, according to predefined formulas. These indices provide integrated measures of metabolic and renal function and were included in the subsequent analyses. To evaluate their associations with MASLD and liver fibrosis, all indices were categorized into quartiles (Supplementary Table 1).

### Evaluation of Hepatic Fat Accumulation and Fibrotic Changes

Hepatic steatosis and fibrosis assessments were performed using VCTE. Participants were instructed to fast for at least 3 hours before the procedure to minimize potential measurement variability. At least 10 valid measurements were obtained for each participant, and only examinations meeting the quality criterion of an interquartile range-to-median ratio of less than 30% were included. CAP values of 248 dB/m or greater were used to define hepatic steatosis based on previously validated cutoffs.^14^^,^^15^ LSM was used to assess fibrosis severity, with values of 9.7 kPa or greater considered indicative of advanced fibrosis.^16^^,^^17^

### Operational Criteria for Metabolic Dysfunction–Associated Steatotic Liver Disease

MASLD was defined by the presence of hepatic steatosis in the absence of alcohol consumption or chronic viral hepatitis, together with at least 1 metabolic risk factor. Metabolic risk factors were defined as follows: (1) increased adiposity defined as a body mass index (BMI) of 25 or greater or central obesity defined as a waist circumference thresholds of 94 cm or greater in men or 80 cm or greater in women; (2) dysglycemia defined as fasting plasma glucose level of 5.6 mmol/L or greater, Glycohemoglobin A1c (HbA1c) level of 5.7% or greater, a diagnosis of type 2 diabetes, or use of glucose-lowering medication; (3) elevated blood pressure, defined as systolic blood pressure of 130 mm Hg or greater, diastolic blood pressure of 85 mm Hg or greater, or current antihypertensive treatment; (4) hypertriglyceridemia, defined as a triglyceride level of 1.70 mmol/L or greater or use of lipid-lowering medication; and (5) reduced HDL-C levels, defined as less than 1.0 mmol/L in men or less than 1.3 mmol/L in women, or use of lipid-lowering therapy.^2^

### Classification of Covariates

Sociodemographic characteristics and health-related behaviors were obtained from standardized self-administered questionnaires and included age, sex, race and ethnicity, education, marital status, income, alcohol consumption, physical activity, and medication use. The poverty income ratio (PIR) was calculated as the ratio of household income to the federal poverty threshold, adjusted for family size and survey cycle, and categorized into 3 levels.^18^ Physical activity was quantified in metabolic equivalent task (MET) minutes per week, calculated as METs multiplied by frequency and duration, with 600 MET min/week or greater considered sufficient physical activity.^19^ Tobacco exposure was assessed based on serum cotinine levels.^20^^,^^21^ Diabetes was defined as a fasting glucose of 7.0 mmol/L or greater, HbA1c level of 6.5% or greater, self-reported diabetes, or use of glucose-lowering therapy.^22^ Hypertension was defined as a blood pressure of 130/80 mm Hg or greater or use of antihypertensive medication.^23^ Alcohol consumption was categorized as abstinent or moderate, defined as 1-2 drinks/day for men and 1 drink/day for women.^24^

### Data Analysis

Continuous data are presented as mean ± SD and categorical data as percentages. Survey-weighted *t*-tests and chi-square tests were used to compare continuous and categorical variables between groups, respectively. Associations between urinary metabolic biomarkers and CAP and LSM were initially evaluated using linear regression. Three models were constructed: model 1, unadjusted; model 2, adjusted for demographic and socioeconomic characteristics; and model 3, further adjusted for smoking, alcohol, diabetes, and hypertension. Logistic regression was used to evaluate associations with MASLD and liver fibrosis, with subgroup analyses stratified by age and sex. Potential nonlinear associations were evaluated using Restricted Cubic Spline (RCS) modeling, whereas propensity score matching was used to balance covariates between groups. Logistic regression analyses were performed in the matched sample to assess the robustness of the observed associations. For threshold analyses, biomarkers were standardized as Z-scores, optimal cutoffs were determined, and participants were classified as high- or low-risk groups for survey-weighted logistic regression. Receiver operating characteristic (ROC) curves and area under the curve (AUROC) were used to evaluate predictive performance. Analyses accounted for complex survey design and weighting and were performed using R 4.1.0 (R Foundation for Statistical Computing, Vienna, Austria) and STATA 16.0 (StataCorp LLC, College Station, TX, USA). No generative AI or large language model tools were used in the conduct of this study, data analysis, or preparation of the manuscript.

## Results

### Demographic and Clinical Features of Study Participants

The analytic cohort comprised 3496 participants. Participants with MASLD were older and predominantly male. Significant differences were observed in race and ethnicity, marital status, smoking status, physical activity, diabetes, hypertension, and BMI (*P* < .05). Regarding metabolic parameters, participants with MASLD had greater waist circumference and higher triglyceride, fasting glucose levels, and HbA1c levels, as well as lower HDL-C. Total cholesterol and low-density lipoprotein cholesterol levels did not differ significantly between the groups. Urinary biomarkers SUACR, UHR, and UAR levels were higher among participants with MASLD, whereas UACR showed a modest increase and BUN/Cr remained unchanged. In the analysis stratified by liver fibrosis status, participants with fibrosis were older and differed significantly from those without fibrosis in sex, education, PIR, physical activity, diabetes, hypertension, and BMI (*P* < .05). The participants with fibrosis also had greater waist circumference and higher triglyceride, glucose, and HbA1c levels, along with lower HDL-C. SUACR, UHR, and UACR were higher among participants with fibrosis, whereas BUN/Cr and UAR showed no significant differences between groups. Complete data are provided in Supplementary Table 2.

### Associations Between Urinary Metabolic Indicators with Controlled Attenuation Parameter and Liver Stiffness Measurement

Table 1 presents the associations between urinary metabolic indicators and CAP. In unadjusted analyses (model 1), SUACR, UHR, and UAR showed positive associations with CAP across quartiles (all *P* < .01). These associations remained statistically significant after sequential adjustment for demographic, socioeconomic, and clinical covariates in models 2 and 3. UACR and BUN/Cr showed weaker and less consistent associations, with statistical significance largely reduced and no longer evident in the fully adjusted models. Similar patterns were observed for LSM (Table 2). Higher SUACR, UACR, UHR, and UAR levels were associated with greater LSM, with stronger associations observed in the higher quartiles. After full adjustment (model 3), however, only UHR and UAR remained significantly associated with LSM, whereas the associations for SUACR, UACR, and BUN/Cr were no longer statistically significant.

### Associations Between Urinary Metabolic Indicators for Metabolic Dysfunction–Associated Steatotic Liver Disease and Liver Fibrosis

Table 3 presents the associations between urinary metabolic indices and MASLD. In unadjusted models (model 1), SUACR, UACR, UHR, and UAR were positively associated with MASLD, with progressively higher odds across quartiles. These associations remained statistically significant after adjustment for potential confounders (all *P* < .05). BUN/Cr was not significantly associated with MASLD in any model. For significant liver fibrosis (Table 4), SUACR, UACR, UHR, and UAR were associated with higher odds in unadjusted analyses. After full adjustment, SUACR, UHR, and UAR remained significantly associated with liver fibrosis, whereas the association with UACR was attenuated and no longer statistically significant. BUN/Cr remained nonsignificant across all models.

The diagnostic performance of urinary metabolic indicators for MASLD and liver fibrosis is presented in Supplementary Table 3, 4 . Overall, these indicators demonstrated modest discriminative ability. For MASLD, UHR had the highest discriminative performance, followed by SUACR and UAR, each showing moderate discrimination. In contrast, UACR demonstrated minimal discriminative ability, with an AUROC close to that expected for a noninformative marker, whereas BUN/Cr provided only modest predictive performance (Figure 2 and Supplementary Table 3). For liver fibrosis, UACR demonstrated the strongest discriminative performance among the evaluated biomarkers, with an AUROC approaching a clinically acceptable level. UAR and BUN/Cr showed modest discriminative ability, whereas SUACR and UHR demonstrated relatively weaker discriminative performance (Supplementary Table 4). The RCS analyses based on the fully adjusted model demonstrated significant associations between urinary metabolic indicators and both MASLD and liver fibrosis. For all evaluated indicators, the overall associations and tests for nonlinearity were statistically significant (all *P* < .05) (Figures 3 and 4).

### Subgroup-Specific and Robustness Analyses

Figure 5 presents the results of subgroup-specific multivariable analyses. Overall, the associations between urinary metabolic indicators and both MASLD and liver fibrosis were generally consistent across subgroups, with no substantial evidence of effect modification. Threshold effect analyses identified significant nonlinear associations for several indicators (Supplementary Table 5). For MASLD, SUACR, UHR, and UAR demonstrated nonlinear relationships (*P* for nonlinear < .05), with stronger associations below the identified thresholds that attenuated above the thresholds. In contrast, UACR demonstrated a linear association (*P* > .05). For liver fibrosis, only UHR demonstrated a significant nonlinear association (*P* < .05), with risk increasing sharply below the identified threshold and then plateauing. No significant nonlinear associations were observed for SUACR and UAR. As a sensitivity analysis, logistic regression was performed in a propensity score–matched cohort. The results were consistent with those of the primary analyses, supporting the robust of the observed associations (Supplementary Tables 6-8).

## Discussion

Evidence from population-based studies linking urinary metabolic markers with VCTE-assessed liver fibrosis remains limited, and a thorough evaluation of multiple kidney- and metabolism-related composite indicators is lacking.^12^^,^^13^ Using nationally representative NHANES data with standardized VCTE measurements, this study comprehensively examined the associations of urinary metabolic markers with hepatic steatosis, MASLD, and liver fibrosis. The results indicate that urinary metabolic indicators, particularly SUACR, UACR, UHR, and UAR, were consistently associated with a higher likelihood of MASLD across multiple models, whereas BUN/Cr was not significantly associated with MASLD. In contrast, associations with liver fibrosis were more selective, with SUACR, UHR, and UAR demonstrating robust independent associations after full adjustment. These findings were further supported by subgroup, spline, and propensity score–matched analyses, supporting the consistency of the observed associations. The identification of nonlinear and threshold-dependent patterns suggests that these indicators may be associated with different risk profiles across exposure levels rather than following simple linear relationships. Overall, by jointly evaluating multiple composite metabolic indicators within a unified framework, this study extends the existing evidence and provides a more integrated perspective on the role of systemic metabolic dysregulation in MASLD and its progression to hepatic fibrosis.

The observed associations between urinary metabolic indicators and hepatic outcomes are biologically plausible and may reflect multiple interrelated pathophysiological processes involving purine metabolism, renal function, lipid homeostasis, and systemic inflammation. In the context of MASLD, several complementary mechanisms may contribute to these associations. First, UA-related indicators (e.g., SUACR, UHR, and UAR) reflect enhanced purine turnover and are closely associated with oxidative stress, because elevated UA levels can promote reactive oxygen species generation and mitochondrial dysfunction, thereby contributing to hepatocellular injury and hepatic steatosis.^25^ Hyperuricemia contributes to the pathogenesis of MASLD by impairing insulin-mediated signaling and promoting systemic metabolic dysregulation.^26^ Moreover, UA directly promotes hepatic fat accumulation by activating lipogenesis-related transcription factors and triggering endoplasmic reticulum stress in hepatocytes.^27^ Composite indicators incorporating HDL-C, such as UHR, capture the imbalance between proinflammatory and anti-inflammatory pathways, because reduced HDL-C weakens its protective effects on cholesterol efflux and vascular inflammation.^28^ In addition, renal-related components such as creatinine and albumin (reflected in SUACR, UACR, and UAR) may serve as indicators of early microvascular dysfunction and endothelial injury. These processes are increasingly recognized as contributors to hepatic steatosis through systemic metabolic crosstalk.^29^

Regarding liver fibrosis, the underlying biological mechanisms may reflect the convergence of persistent inflammatory activation and progressive extracellular matrix remodeling. Experimental evidence suggests that elevated UA can directly promote hepatic stellate cell proliferation and activation, thereby accelerating collagen deposition and fibrogenesis.^30^ In parallel, UA has been shown to induce hepatic injury through activation of the NLRP3 inflammasome, a key mediator of innate immune signaling that promotes chronic inflammation and facilitates the progression from steatosis to steatohepatitis and fibrosis.^31^ Inflammasome-mediated signaling further amplifies fibrogenic responses by promoting cytokine release and hepatic stellate cell activation, underscoring the potential role of NLRP3 signaling in liver fibrosis progression.^31^ From a circulatory perspective, renal dysfunction–related indicators such as UACR and BUN/Cr may reflect systemic microvascular dysfunction and altered perfusion. These disturbances can exacerbate intrahepatic hypoxia, a well-recognized trigger of fibrogenic signaling pathways and tissue remodeling.^32^ Meanwhile, indicators centered on albumin (e.g., UAR) provide information on the link between systemic inflammatory activity and nutritional health, with reduced albumin levels reflecting compromised antioxidant function and enhanced inflammatory burden, both of which contribute to the advancement of fibrosis.^33^ Furthermore, dyslipidemia-related mechanisms, partially reflected by indicators such as UHR, may promote hepatocellular lipotoxicity, resulting in oxidative stress, apoptosis, and subsequent activation of fibrogenic pathways. These processes facilitate the progression from simple steatosis to advanced fibrotic remodeling.^34^ Collectively, these findings suggest that urinary metabolic indicators should not be interpreted solely as markers of renal function but rather as integrative surrogates of systemic metabolic dysregulation, inflammatory activation, and microvascular dysfunction, which may collectively contribute to the development of MASLD and subsequent liver fibrosis.

This study is among the first to assess the associations between urinary metabolic indicators and MASLD and liver fibrosis at the population level using VCTE. Limitations include the cross-sectional design, which does not allow determination of temporal relationships or causal conclusions. Urinary biomarkers provide a snapshot of relatively stable metabolic and renal profiles and may not capture dynamic inflammatory changes across different stages of disease; therefore, causal inferences cannot be established. Although VCTE provides noninvasive assessment of liver steatosis and fibrosis, the absence of liver biopsy, longitudinal biomarker measurements, and follow-up limits the assessment of disease progression over time. Potential residual confounding cannot be completely excluded. Despite these limitations, the observed associations remained consistent across multiple analytic models. Future longitudinal studies incorporating repeated biomarker measurements and mechanistic investigations are warranted to elucidate the potential relationship of systemic metabolic dysregulation in the development of MASLD and liver fibrosis.

## Conclusion

This nationally representative cross-sectional study demonstrated significant associations between urinary metabolic markers and liver health assessed by VCTE. SUACR, UACR, UHR, and UAR were consistently associated with MASLD, whereas SUACR, UHR, and UAR remained significantly associated with liver fibrosis after multivariable adjustment. Although these markers demonstrated moderate predictive performance, they reflect systemic metabolic and renal disturbances relevant to the pathogenesis of liver disease. By evaluating multiple urinary composite indicators within a unified framework, this study provides novel population-level evidence. Prospective studies are needed to validate these findings and determine the potential utility of these biomarkers for clinical risk assessment and longitudinal monitoring.

## Supplementary Materials

Supplementary Material

## Figures and Tables

**Figure 1.**
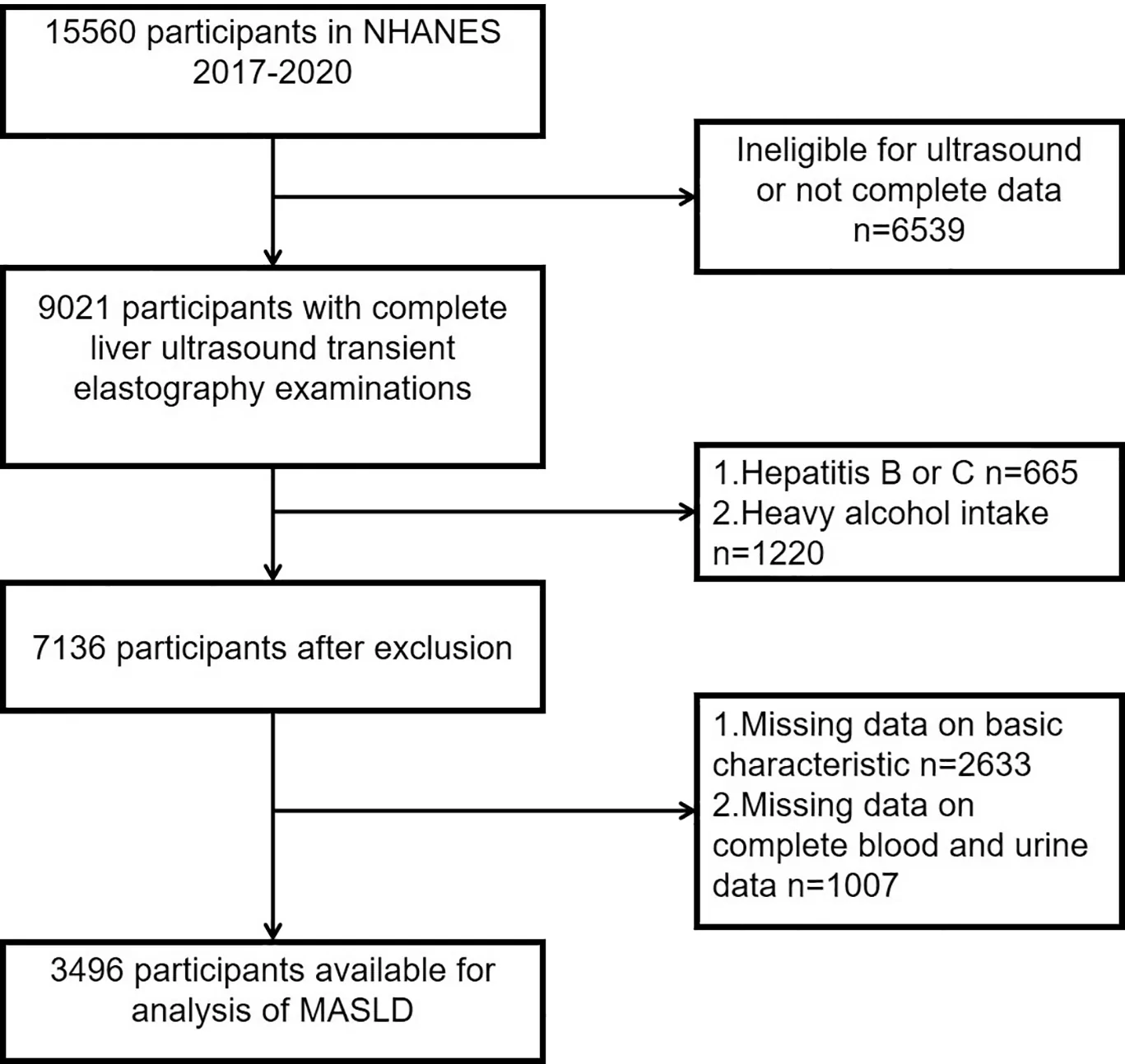
Flow diagram depicting participant inclusion and exclusion. MASLD, metabolic dysfunction**–**associated steatotic liver disease; NHANES, National Health and Nutrition Examination Survey.

**Figure 2.**
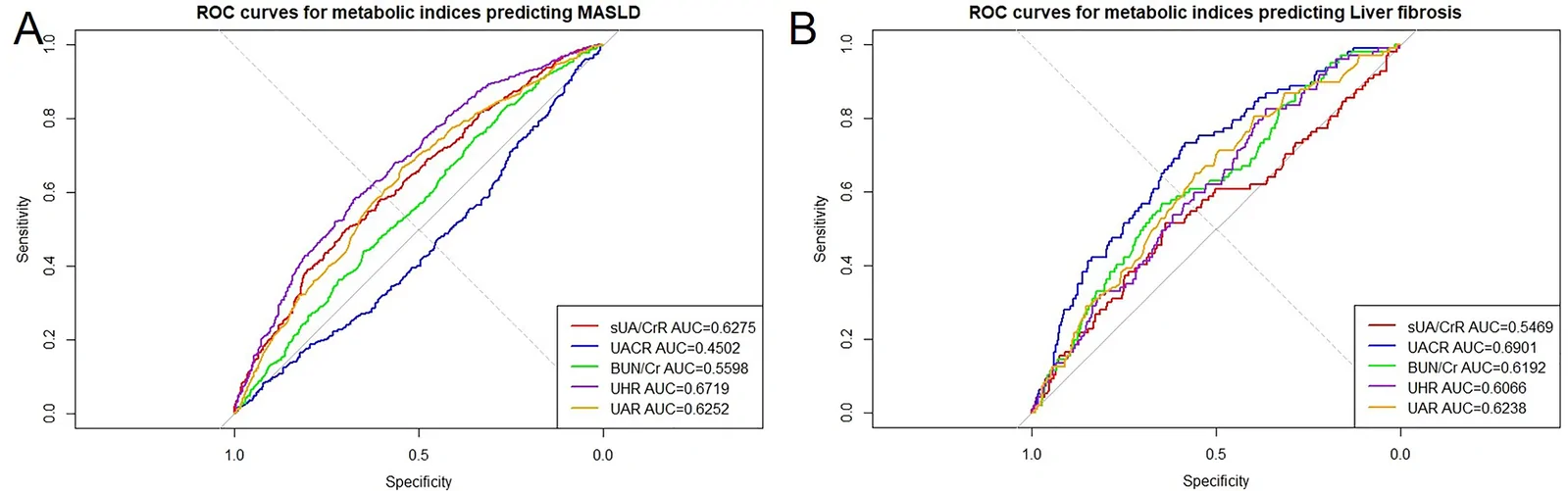
ROC analyses of urinary metabolic indicators for (A) MASLD and (B) advanced liver fibrosis detection. AUC, area under the curve; BUN/Cr, blood urea nitrogen-to-creatinine ratio; MASLD, metabolic dysfunction**–**associated steatotic liver disease; ROC, receiver operating characteristic; SUACR, serum uric acid-to-creatinine ratio; UACR, urinary albumin-to-creatinine ratio; UAR, uric acid-to-albumin ratio; UHR, uric acid-to-high-density lipoprotein cholesterol ratio.

**Figure 3.**
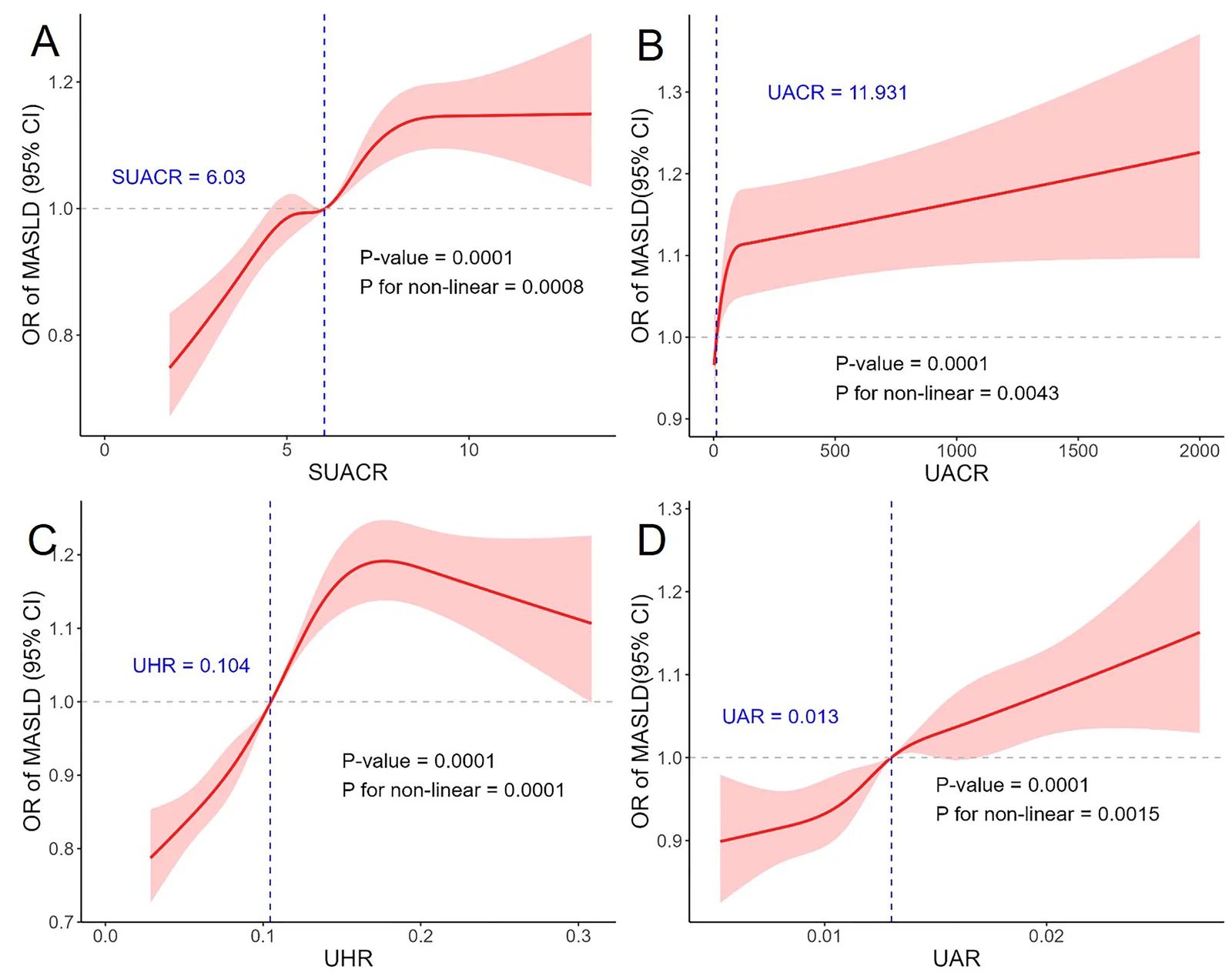
RCS models depicting associations of MASLD and (A) SUACR, (B) UACR, (C) UHR, (D) UAR. MASLD, metabolic dysfunction**–**associated steatotic liver disease; SUACR, serum uric acid-to-creatinine ratio; UACR, urinary albumin-to-creatinine ratio; UAR, uric acid-to-albumin ratio; UHR, uric acid-to-high-density lipoprotein cholesterol ratio.

**Figure 4.**
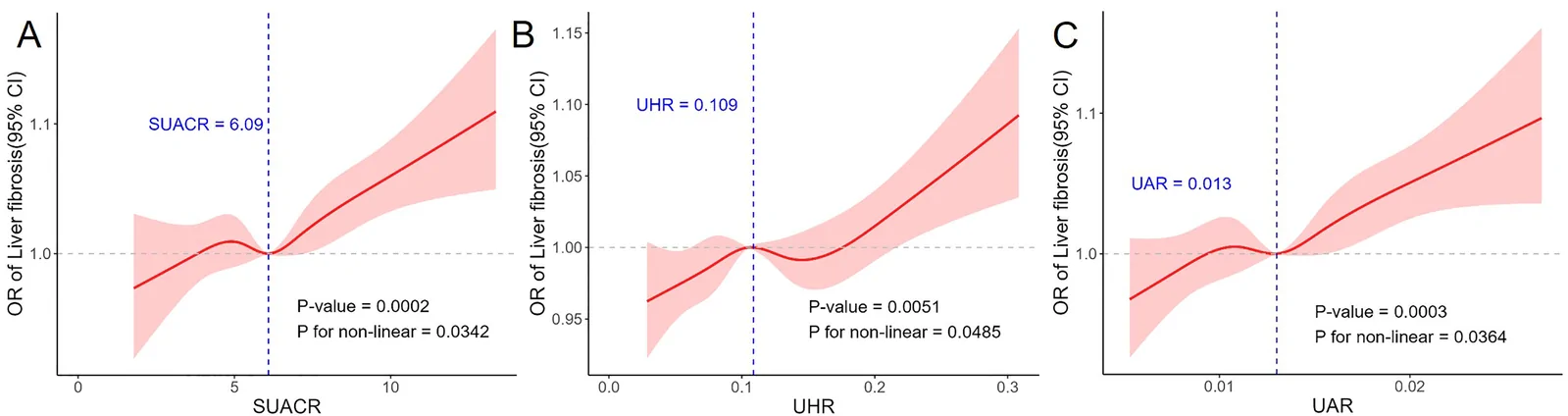
RCS models depicting associations of liver fibrosis and (A) SUACR, (B) UHR, (C) UAR. SUACR, serum uric acid-to-creatinine ratio; UAR, uric acid-to-albumin ratio; UHR, uric acid-to-high-density lipoprotein cholesterol ratio.

**Figure 5.**
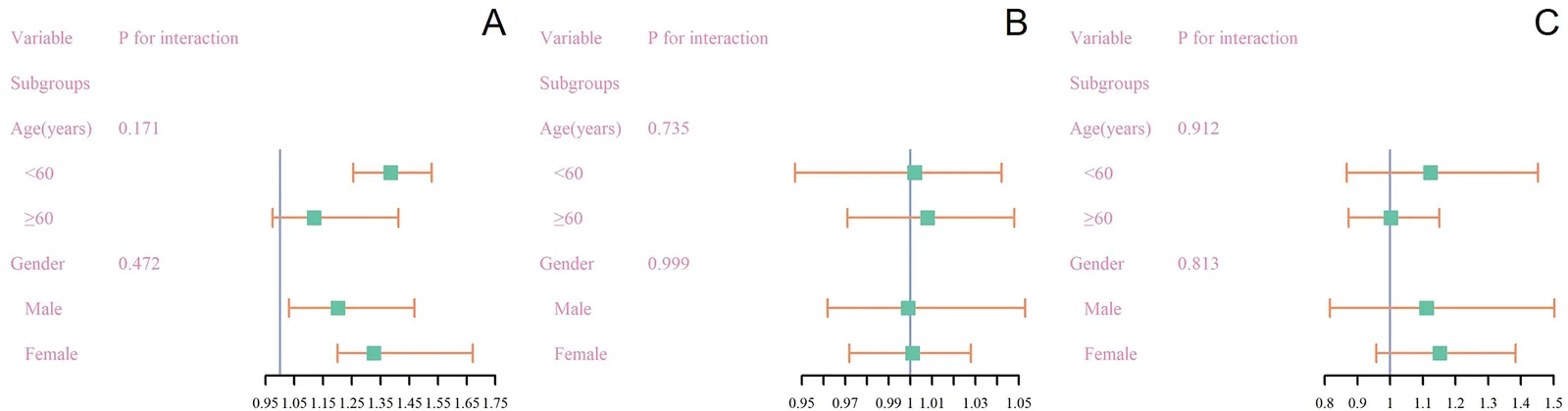
Stratified analyses of MASLD with (A) SUACR and (B) UACR and liver fibrosis with (C) SUACR. MASLD, metabolic dysfunction**–**associated steatotic liver disease; SUACR, serum uric acid-to-creatinine ratio; UACR, urinary albumin-to-creatinine ratio.

**Table 1.** Correlations of Urinary Metabolic Indicators with CAP

Index	Model	Q1	Q2		Q3		Q4	
			*β* (95% CI)	*P*	*β* (95% CI)	*P*	*β* (95% CI)	*P*
SUACR	Model 1	Reference	11.723 (3.450-19.996)	.005	17.920 (9.799-26.040)	<.001	42.545 (34.284- 50.806)	<.001
	Model 2	Reference	8.095 (1.408-14.782)	.018	11.802 (5.042-18.562)	<.001	27.424 (19.554-35.293)	<.001
	Model 3	Reference	9.259 (2.806-15.712)	.005	13.314 (6.916-19.711)	<.001	25.489 (18.041-32.936)	<.001
UACR	Model 1	Reference	0.970 (−7.126 to 9.065)	.814	6.071 (−2.528 to 14.670)	.166	15.134 (6.210-24.057)	<.001
	Model 2	Reference	5.408 (−1.431 to 12.246)	.121	5.239 (−2.077 to 12.555)	.162	10.312 (2.319-18.305)	.011
	Model 3	Reference	1.930 (−4.759 to 8.618)	.572	0.454 (−6.662 to 7.571)	.900	−3.320 (−11.352 to 4.711)	.418
BUN/Cr	Model 1	Reference	5.365 (−3.321 to 14.052)	.226	8.287 (0.348-16.226)	.041	8.881 (0.898-16.865)	.029
	Model 2	Reference	5.390 (−1.185 to 11.965)	.108	7.290 (−0.068 to 14.648)	.052	5.977 (−1.872 to 13.827)	.136
	Model 3	Reference	4.664 (−1.503 to 10.832)	.138	6.045 (−0.894 to 12.984)	.088	0.363 (−6.897 to 7.623)	.922
UHR	Model 1	Reference	26.975 (19.243-34.707)	<.001	39.107 (31.093-47.121)	<.001	71.735 (64.051-79.418)	<.001
	Model 2	Reference	12.506 (5.282-19.731)	<.001	23.662 (16.078-31.247)	<.001	45.488 (37.259-53.716)	<.001
	Model 3	Reference	10.790 (3.954-17.626)	.002	19.862 (12.260-27.464)	<.001	37.399 (29.266-45.531)	<.001
UAR	Model 1	Reference	19.948 (11.559-28.336)	<.001	35.556 (27.633-43.479)	<.001	52.605 (44.764-60.445)	<.001
	Model 2	Reference	5.044 (−1.935 to 12.023)	.157	14.053 (6.434-21.672)	<.001	23.701 (16.149-31.252)	<.001
	Model 3	Reference	5.022 (−1.465 to 11.509)	.129	14.204 (6.803-21.605)	<.001	20.952 (13.799-28.105)	<.001

BUN/Cr, blood urea nitrogen-to-creatinine ratio; CAP, controlled attenuation parameter; SUACR, serum uric acid-to-creatinine ratio; UACR, urinary albumin-to-creatinine ratio; UAR, uric acid-to-albumin ratio; UHR, uric acid-to-high-density lipoprotein cholesterol ratio.

**Table 2.** Correlations of Urinary Metabolic Indicators with LSM

Index	Model	Q1	Q2		Q3		Q4	
			*β* (95% CI)	*P*	*β* (95% CI)	*P*	*β* (95% CI)	*P*
SUACR	Model 1	Reference	0.095 (−0.474 to 0.664)	.743	0.104 (−0.481 to 0.689)	.727	0.711 (0.163-1.258)	.011
	Model 2	Reference	0.037 (−0.518 to 0.592)	.896	0.013 (−0.577 to 0.602)	.966	0.429 (−0.088 to 0.947)	.104
	Model 3	Reference	0.080 (−0.446 to 0.606)	.765	0.079 (−0.519 to 0.678)	.795	0.376 (−0.117 to 0.869)	.135
UACR	Model 1	Reference	0.034 (−0.609 to 0.541)	.907	0.100 (−0.436 to 0.636)	.715	0.985 (0.364-1.607)	.002
	Model 2	Reference	0.153 (−0.410 to 0.717)	.594	0.120 (−0.407 to 0.647)	.656	0.857 (0.245-1.469)	.006
	Model 3	Reference	0.085 (−0.490 to 0.660)	.771	0.021 (−0.528 to 0.571)	.939	0.448 (−0.136 to 1.033)	.133
BUN/Cr	Model 1	Reference	0.065 (−0.570 to 0.700)	.842	0.023 (−0.556 to 0.602)	.938	0.164 (−0.446 to 0.774)	.599
	Model 2	Reference	0.181 (−0.462 to 0.824)	.581	0.225 (−0.340 to 0.791)	.435	0.519 (−0.044 to 1.083)	.071
	Model 3	Reference	0.139 (−0.486 to 0.763)	.663	0.150 (−0.411 to 0.711)	.601	0.295 (−0.235 to 0.824)	.275
UHR	Model 1	Reference	0.900 (0.484-1.316)	<.001	1.050 (0.650-1.449)	<.001	1.977 (1.329-2.624)	<.001
	Model 2	Reference	0.431 (−0.028 to 0.890)	.066	0.460 (0.053-0.867)	.027	1.022 (0.406-1.638)	.001
	Model 3	Reference	0.392 (−0.045 to 0.829)	.078	0.332 (−0.076 to 0.740)	.111	0.711 (0.110-1.312)	.020
UAR	Model 1	Reference	0.868 (0.399-1.337)	<.001	1.068 (0.598-1.537)	<.001	1.679 (1.114-2.245)	<.001
	Model 2	Reference	0.362 (−0.076 to 0.801)	.105	0.382 (−0.068 to 0.832)	.096	0.757 (0.187-1.326)	.009
	Model 3	Reference	0.392 (−0.035 to 0.818)	.072	0.419 (−0.026 to 0.864)	.065	0.698 (0.136-1.261)	.015

BUN/Cr, blood urea nitrogen-to-creatinine ratio; LSM, liver stiffness measurement; SUACR, serum uric acid-to-creatinine ratio; UACR, urinary albumin-to-creatinine ratio; UAR, uric acid-to-albumin ratio; UHR, uric acid-to-high-density lipoprotein cholesterol ratio.

**Table 3.** Correlations of Urinary Metabolic Indicators with MASLD

Index	Model	Q1	Q2		Q3		Q4	
			OR (95% CI)	*P*	OR (95% CI)	*P*	OR (95% CI)	*P*
SUACR	Model 1	Reference	1.289 (0.959-1.733)	.092	1.504 (1.116-2.029)	.007	3.616 (2.632-4.967)	<.001
	Model 2	Reference	1.262 (0.893-1.784)	.186	1.408 (1.002-1.981)	.048	2.849 (1.950-4.161)	<.001
	Model 3	Reference	1.370 (0.953-1.971)	.090	1.564 (1.096-2.232)	.014	2.644 (1.833-3.814)	<.001
UACR	Model 1	Reference	1.092 (0.820-1.455)	.547	1.274 (0.939-1.728)	.119	1.358 (1.010-1.826)	.043
	Model 2	Reference	1.202 (0.858-1.684)	.285	1.341 (0.955-1.881)	.090	2.574 (1.735-3.819)	<.001
	Model 3	Reference	1.375 (0.962-1.965)	.081	1.409 (0.981-2.026)	.064	2.285 (1.496-3.488)	<.001
BUN/Cr	Model 1	Reference	0.908 (0.674-1.224)	.528	0.843 (0.631-1.125)	.245	0.859 (0.637-1.159)	.321
	Model 2	Reference	1.366 (0.985-1.896)	.062	1.375 (0.946-1.999)	.095	1.202 (0.833-1.734)	.325
	Model 3	Reference	1.221 (0.868-1.717)	.252	1.089 (0.755-1.573)	.648	0.848 (0.586-1.228)	.383
UHR	Model 1	Reference	2.282 (1.708-3.048)	<.001	3.650 (2.676-4.978)	<.001	9.577 (6.786-13.518)	<.001
	Model 2	Reference	1.596 (1.138-2.237)	.007	3.057 (2.159-4.328)	<.001	6.413 (4.250-9.677)	<.001
	Model 3	Reference	1.623 (1.156-2.278)	.005	2.830 (1.953-4.103)	<.001	4.914 (3.222-7.494)	<.001
UAR	Model 1	Reference	1.917 (1.429-2.573)	<.001	3.257 (2.420-4.383)	<.001	4.983 (3.647-6.806)	<.001
	Model 2	Reference	1.359 (0.986-1.872)	.061	2.074 (1.459-2.948)	<.001	2.613 (1.833-3.727)	<.001
	Model 3	Reference	1.551 (1.103-2.183)	.012	1.741 (1.220-2.484)	.002	1.719 (1.198-2.466)	.003

BUN/Cr, blood urea nitrogen-to-creatinine ratio; MASLD, metabolic dysfunction–associated steatotic liver disease; SUACR, serum uric acid-to-creatinine ratio; UACR, urinary albumin-to-creatinine ratio; UAR, uric acid-to-albumin ratio; UHR, uric acid-to-high-density lipoprotein cholesterol ratio.

**Table 4.** Correlations of Urinary Metabolic Indicators with Liver Fibrosis

Index	Model	Q1	Q2		Q3		Q4	
			OR (95% CI)	*P*	OR (95% CI)	*P*	OR (95% CI)	*P*
SUACR	Model 1	Reference	1.034 (0.571-1.872)	.912	0.857 (0.487-1.509)	.593	2.460 (1.471-4.114)	.001
	Model 2	Reference	0.945 (0.522-1.712)	.852	0.760 (0.423-1.368)	.362	1.904 (1.124-3.226)	.017
	Model 3	Reference	0.947 (0.494-1.814)	.869	0.805 (0.436-1.487)	.488	1.885 (1.106-3.213)	.019
UACR	Model 1	Reference	0.987 (0.497-1.960)	.970	1.929 (1.080-3.447)	.027	4.150 (2.354-7.316)	<.001
	Model 2	Reference	1.020 (0.498-2.091)	.957	1.611 (0.882-2.941)	.121	3.200 (1.726-5.933)	<.001
	Model 3	Reference	0.889 (0.411-1.920)	.764	1.302 (0.693-2.445)	.412	1.901 (0.974-3.713)	.060
BUN/Cr	Model 1	Reference	1.277 (0.730-2.233)	.391	1.159 (0.625-2.151)	.639	1.959 (1.124-3.412)	.018
	Model 2	Reference	1.363 (0.768-2.419)	.291	1.282 (0.644-2.552)	.479	1.283 (0.707-2.330)	.413
	Model 3	Reference	1.351 (0.751-2.432)	.315	1.213 (0.575-2.557)	.612	1.740 (0.938-3.224)	.079
UHR	Model 1	Reference	5.263 (2.497-11.096)	<.001	5.067 (2.469-10.399)	<.001	8.010 (4.070-15.764)	<.001
	Model 2	Reference	3.635 (1.671-7.911)	<.001	3.308 (1.554-7.042)	<.001	3.839 (1.692-8.711)	<.001
	Model 3	Reference	3.519 (1.620-7.646)	.001	2.703 (1.270-5.754)	.009	2.726 (1.249-5.948)	.012
UAR	Model 1	Reference	1.872 (0.885-3.960)	.101	2.225 (1.158-4.274)	.016	4.220 (2.311-7.706)	<.001
	Model 2	Reference	1.218 (0.728-2.037)	.452	1.434 (0.853-2.410)	.174	1.786 (1.094-2.916)	.020
	Model 3	Reference	1.222 (0.726-2.057)	.451	1.479 (0.887-2.464)	.133	1.663 (1.024-2.701)	.039

BUN/Cr, blood urea nitrogen-to-creatinine ratio; SUACR, serum uric acid-to-creatinine ratio; UACR, urinary albumin-to-creatinine ratio; UAR, uric acid-to-albumin ratio; UHR, uric acid-to-high-density lipoprotein cholesterol ratio.

## Data Availability

The data that support the findings of this study are available on request from the corresponding author.
