# Supplementary material for: Associations Between Urinary Metabolic Indicators and Metabolic Dysfunction–Associated Steatotic Liver Disease and Liver Fibrosis: Evidence from National Health and Nutrition Examination Survey 2017-2020
Source: Turk J Gastroenterol. 2026 Sep 8;37(10):1077–88. doi: 10.5152/tjg.2026.26228 (PMC13752727; doi:10.5152/tjg.2026.26228)
Supplement: Supplementary file 1 [file supplementary_material.pdf]

Supplementary Table 1. Quartile cutoffs of urinary metabolic indicators.

Index	Quartiles 1	Quartiles 2	Quartiles 3	Quartiles 4
SUA/Cr	<5.0538	5.0538-6.0526	6.0526-7.2414	≥.7.2414
UACR	<4.79	4.79-7.59	7.59-15.2525	≥6.15.2525
BUN/Cr	<13.6364	13.6364-16.6667	16.6667-20.6612	≥20.6612
UHR	<0.077	0.077-0.105	0.105-0.141	≥0.141
UAR	<0.0108	0.0108-0.013	0.013-0.0155	≥0.0155

BUN/Cr, Blood urea nitrogen-to-creatinine ratio; SUA/Cr, Serum uric acid-to-creatinine ratio; UACR, Urinary albumin-to-creatinine ratio; UAR, Uric acid-to-albumin ratio; UHR, Uric acid-to-high-density lipoprotein cholesterol ratio.

Supplementary Table 2. Descriptive baseline characteristics of the study cohort.

	Non-MASLD (n=1427)	MASLD (n=2069)	*P*-value	Non-Liver fibrosis (n=3275)	Liver fibrosis (n=221)	*P*-value
Age (year)	48.29(0.70)	55.39(0.50)	<.001	52.04(0.42)	60.14(0.73)	.045
Gender(n,%)			<.001			.008
Male	676(47.37)	1095(52.92)		1640(50.08)	131(59.28)	
Female	751(52.63)	974(47.08)		1635(49.92)	90(40.72)	
Race(n,%)			<.001			.057
Mexican American	92(6.45)	269(13.00)		335(10.23)	26(11.76)	
Other Hispanic	129(9.04)	211(10.20)		311(9.50)	29(13.12)	
Non-Hispanic white	505(35.39)	815(39.39)		1228(37.50)	92(41.63)	
Non-Hispanic black	428(29.99)	430(20.78)		811(24.76)	47(21.27)	
Other	273(19.13)	344(16.63)		590(18.01)	27(12.22)	
Education level(n,%)			.282			.003
Less than high school	217(15.21)	377(18.22)		550(16.79)	44(19.91)	
High school or equivalent	309(21.65)	469(22.67)		712(21.74)	66(29.86)	
Above high school	901(63.14)	1223(59.11)		2013(61.47)	111(50.23)	
Marital status (n, %)			<.001			.278
Married/cohabitant	803(56.27)	1325(64.04)		1989(60.73)	139(62.90)	
Widowed/divorced/separated	308(21.58)	484(23.39)		738(22.53)	54(24.43)	
Never married	316(22.15)	260(12.57)		548(16.73)	28(12.67)	
Poverty income ratio (n, %)			.269			.002
<1.30	381(26.70)	523(25.28)		844(25.77)	60(27.15)	
1.30-3.50	532(37.28)	827(39.97)		1253(38.26)	106(47.96)	
>3.50	514(36.02)	719(34.75)		1178(35.97)	55(24.89)	
Drinking status (n, %)			.239			.981
Non	634(44.43)	961(46.45)		1494(45.62)	101(45.70)	
Low to moderate	793(55.57)	1108(53.55)		1781(54.38)	120(54.30)	
Smoking habits (n, %)			<.001			.703
Never	526(36.86)	843 (40.74)		1280(39.08)	89(40.27)	
Moderate	577(40.43)	890 (43.02)		1372(41.89)	95(42.99)	
Heavy	324(22.71)	336(16.24)		623(19.02)	37(16.74)	

	Non-MASLD (n=1427)	MASLD (n=2069)	*P*-value	Non-Liver fibrosis (n=3275)	Liver fibrosis (n=221)	*P*-value
Physical activity (n, %)			<.001			.006
Never	313(21.94)	576(27.84)		815(24.89)	74(33.48)	
Insufficient	164(11.49)	265(12.81)		398(12.15)	31(14.03)	
Constant	950(66.57)	1228(59.35)		2062(62.96)	116(52.49)	
Diabetes(n, %)			<.001			<.001
Yes	147(10.30)	677(32.72)		700(21.37)	124(56.11)	
No	1280(89.70)	1392(67.28)		2575(78.63)	97(43.89)	
Hypertension(n, %)			<.001			<.001
Yes	649(45.48)	1424(68.83)		1889(57.68)	184(83.26)	
No	778(54.52)	645(31.17)		1386(42.32)	37(16.74)	
BMI (kg/m2)			<.001			<.001
<28	1031(72.25)	563(27.21)		1559(47.60)	35(15.84)	
≥5(	396(27.75)	1506(72.79)		1716(52.40)	186(84.16)	
WC(cm)	89.72(0.50)	107.82(0.47)	<.001	99.39(0.40)	118.30(0.93)	<.001
TG (mmol/L)	0.90(0.02)	1.41(0.03)	<.001	1.19(0.02)	1.38(0.07)	<.001
TC (mmol/L)	4.73(0.04)	4.79(0.03)	.557	4.79(0.04)	4.40(0.11)	<.001
LDL (mmol/L)	2.82(0.05)	2.88(0.04)	.395	2.87(0.04)	2.52(0.09)	.395
HDL (mmol/L)	1.50(0.01)	1.27(0.01)	<.001	1.37(0.01)	1.25(0.04)	<.001
FPG(mmol/L)	5.67(0.04)	6.77(0.07)	<.001	6.23(0.05)	7.84(0.11)	<.001
Hb1Ac(%)	5.55(0.02)	6.16(0.04)	<.001	5.85(0.03)	6.91(0.09)	<.001
SUA/Cr	5.87(0.04)	6.60(0.04)	<.001	6.23(0.03)	6.75(0.05)	<.001
UACR	48.84(7.19)	47.74(5.04)	.032	44.56(4.99)	75.09(6.78)	<.001
BUN/Cr	17.06(0.02)	17.79(0.04)	.246	17.42(0.10)	18.67(0.25)	.329
UHR	0.0948(0.0011)	0.1265(0.0011)	<.001	0.1117(0.0009)	0.1407(0.0018)	<.001
UAR	0.0123(0.0001)	0.0142(0.0001)	<.001	0.0133(0.0001)	0.0155(0.0001)	.162

BMI, Body mass index; BUN/Cr, Blood urea nitrogen-to-creatinine ratio; FPG, Fasting plasma glucose; HbA1c, Glycohemoglobin A1c; HDL, High-density lipoprotein cholesterol; LDL, Low-density lipoprotein cholesterol; MASLD, Metabolic dysfunction–associated steatotic liver disease; SUA/Cr, Serum uric acid-to-creatinine ratio; TC, Total cholesterol; TG, Triglyceride; UACR, Urinary albumin-to-creatinine ratio; UAR, Uric acid-to-albumin ratio; UHR, Uric acid-to-high-density lipoprotein cholesterol ratio; WC, Waist circumference.

Supplementary Table 3. Screening accuracy of urinary metabolic indicators for MASLD.

Index	AUROC	Sensitivity	Specificity	95% CI	Positive predictive value	Negative predictive value	Threshold
SUA/Cr	0.6275	0.4905	0.7074	0.5991-0.6561	0.7484	0.4389	0.6584
UACR	0.4502	0.9550	0.0549	0.4205-0.4803	0.6420	0.4078	0.6334
BUN/Cr	0.5598	0.8201	0.2765	0.5299-0.5896	0.6680	0.4642	0.6215
UHR	0.6719	0.5794	0.6773	0.6442-0.6995	0.7611	0.4757	0.6295
UAR	0.6252	0.6663	0.5461	0.5964-0.6541	0.7226	0.4797	0.6112

BUN/Cr, Blood urea nitrogen-to-creatinine ratio; MASLD, Metabolic dysfunction–associated steatotic liver disease; SUA/Cr, Serum uric acid-to-creatinine ratio; UACR, Urinary albumin-to-creatinine ratio; UAR, Uric acid-to-albumin ratio; UHR, Uric acid-to-high-density lipoprotein cholesterol ratio.

Supplementary Table 4. Screening accuracy of urinary metabolic indicators for liver fibrosis.

Index	AUROC	Sensitivity	Specificity	95% CI	Positive predictive value	Negative predictive value	Threshold
SUA/Cr	0.5469	0.5154	0.6376	0.4821-0.6117	0.0859	0.9521	0.0517
UACR	0.6901	0.7319	0.5858	0.6367-0.7434	0.1045	0.9706	0.0503
BUN/Cr	0.6192	0.5670	0.6491	0.5629-0.6756	0.0964	0.9577	0.0495
UHR	0.6066	0.8247	0.3658	0.5518-0.6613	0.0791	0.9693	0.0433
UAR	0.6238	0.6494	0.5579	0.5682-0.6793	0.0884	0.9601	0.0487

BUN/Cr, Blood urea nitrogen-to-creatinine ratio; SUA/Cr, Serum uric acid-to-creatinine ratio; UACR, Urinary albumin-to-creatinine ratio; UAR, Uric acid-to-albumin ratio; UHR, Uric acid-to-high-density lipoprotein cholesterol ratio.

Supplementary Table 5. Urinary metabolic indicators: threshold-dependent effects on MASLD and fibrosis.

Variable	Breakpoint	OR_before (95% CI)	OR_after (95% CI)	*P*_nonlinear
MASLD				
SUA/Cr	-1.1788	9.405 (1.732% CI))in	1.414 (1.158% CI))i	<.0001
UACR	0.3036	1.205 (0.857(0.857)	0.867 (0.669(0.669)	.3893
UHR	0.7834	2.378 (1.681) CI))i	0.845 (0.475) CI))i	<.0001
UAR	0.0454	2.305 (1.556) CI))i	1.041 (0.797) CI))i	<.0001
Liver fibrosis				
SUA/Cr	-0.1736	0.955 (0.474–1.924)	1.400 (0.941–1.924)	0.2429
UHR	-0.4426	7.774 (1.910–1.924)n	0.796 (0.554–1.924)	0.0012
UAR	1.2318	1.531 (0.925–1.924)	0.714 (0.345–1.924)	0.2321

MASLD, Metabolic dysfunction–associated steatotic liver disease; SUA/Cr, Serum uric acid-to-creatinine ratio; UACR, Urinary albumin-to-creatinine ratio; UAR, Uric acid-to-albumin ratio; UHR, Uric acid-to-high-density lipoprotein cholesterol ratio.

Supplementary Table 6. Post-propensity score matching cohort characteristics.

	Non-MASLD (n=1427)	MASLD (n=1427)	*P*-value	Non-Liver fibrosis (n=488)	Liver fibrosis (n=488)	*P*-value
Age (year)	48.29(0.70)	56.65(0.55)	<.001	58.83(0.52)	60.14(0.73)	.532
Gender(n,%)			<.001			.435
Male	676(47.37)	783(54.87)		139(62.90)	131(59.28)	
Female	751(52.63)	644(45.13)		82(37.10)	90(40.72)	
Race(n,%)			<.001			.285
Mexican American	92(6.45)	212(14.86)		27(12.22)	26(11.76)	
Other Hispanic	129(9.04)	158(11.07)		17(7.69)	29(13.12)	
Non-Hispanic white	505(35.39)	606(42.47)		87(39.37)	92(41.63)	
Non-Hispanic black	428(29.99)	303(21.23)		60(27.15)	47(21.27)	
Other	273(19.13)	148(10.37)		30(13.57)	27(12.22)	
Education level(n,%)			.008			.842
Less than high school	217(15.21)	270(18.92)		40(18.10)	44(19.91)	
High school or equivalent	309(21.65)	331(23.20)		49(22.17)	66(29.86)	
Above high school	901(63.14)	826(57.88)		132(59.73)	111(50.23)	
Marital status (n, %)			<.001			.104

	Non-MASLD (n=1427)	MASLD (n=1427)	*P*-value	Non-Liver fibrosis (n=488)	Liver fibrosis (n=488)	*P*-value
Married/cohabitant	803(56.27)	960(67.27)		142(64.25)	139(62.90)	
Widowed/divorced/separated	308(21.58)	335(23.48)		49(22.17)	54(24.43)	
Never married	316(22.15)	132(9.25)		30(13.57)	28(12.67)	
Poverty income ratio (n, %)			.334			.197
<1.30	381(26.70)	360(25.23)		65(29.41)	60(27.15)	
1.30-3.50	532(37.28)	570(39.94)		88(39.82)	106(47.96)	
>3.50	514(36.02)	497(34.83)		68(30.77)	55(24.89)	
Drinking status (n, %)			.452			.502
Non	634(44.43)	655(45.90)		94(42.53)	101(45.70)	
Low to moderate	793(55.57)	772(54.10)		127(57.47)	120(54.30)	
Smoking habits (n, %)			<.001			.879
Never	526(36.86)	622 (43.59)		88(39.82)	89(40.27)	
Moderate	577(40.43)	582 (40.78)		92(41.63)	95(42.99)	
Heavy	324(22.71)	223(15.63)		41(18.55)	37(16.74)	
Physical activity (n, %)			.011			.762
Never	313(21.94)	413(28.94)		71(32.13)	74(33.48)	
Insufficient	164(11.49)	177(12.40)		27(12.22)	31(14.03)	
Constant	950(66.57)	837(58.65)		123(55.66)	116(52.49)	
Diabetes(n, %)			<.001			.924
Yes	147(10.30)	585(41.00)		125(56.56)	124(56.11)	
No	1280(89.70)	842(59.00)		96(43.44)	97(43.89)	
Hypertension(n, %)			<.001			.392
Yes	649(45.48)	1111(77.86)		176(79.64)	184(83.26)	
No	778(54.52)	316(22.14)		45(20.36)	37(16.74)	
BMI (kg/m2)			<.001			.502
<28	1031(72.25)	50(3.50)		30(13.57)	35(15.84)	
≥5(	396(27.75)	1377(96.50)		191(86.43)	186(84.16)	
WC(cm)	89.72(0.50)	113.04(0.50)	<.001	109.08(1.37)	118.30(0.93)	<.001
TG (mmol/L)	0.90(0.02)	1.46(0.03)	<.001	1.31(0.07)	1.38(0.07)	.957
TC (mmol/L)	4.73(0.04)	4.72(0.04)	.748	4.44(0.09)	4.40(0.11)	.793
LDL (mmol/L)	2.82(0.05)	2.83(0.04)	.946	2.60(0.09)	2.52(0.09)	.777
HDL (mmol/L)	1.50(0.01)	1.22(0.01)	<.001	1.24(0.03)	1.25(0.04)	.702
FPG(mmol/L)	5.67(0.04)	7.05(0.09)	<.001	7.16(0.13)	7.84(0.11)	.124
Hb1Ac(%)	5.55(0.02)	6.35(0.05)	<.001	6.37(0.12)	6.91(0.09)	.157
SUA/Cr	5.87(0.04)	6.87(0.07)	<.001	6.76(0.07)	6.75(0.05)	.198
UACR	48.84(7.19)	51.20(11.74)	.702	69.56(5.23)	75.09(6.78)	.652
UHR	0.0948(0.0011)	0.1328(0.0014)	<.001	0.1332(0.0024)	0.1407(0.0018)	.014
UAR	0.0123(0.0001)	0.0146(0.0001)	<.001	0.0145(0.0002)	0.0155(0.0001)	.015

BMI, Body mass index; SUA/Cr, Serum uric acid-to-creatinine ratio; UACR, Urinary albumin-to-creatinine ratio; UAR, Uric acid-to-albumin ratio; UHR, Uric acid-to-high-density lipoprotein cholesterol ratio.

Supplementary Table 7. Adjusted logistic regression for MASLD with urinary metabolic indicators after PSM (Model 3).

		Q2		Q3		Q4	
Index	Q1	OR (95%CI)	*P* value	OR (95%CI)	*P* value	OR (95%CI)	*P* value
SUA/Cr	ref	1.634(0.918-2.908)	.095	2.131(1.254-3.622)	0.005	3.466(1.974-6.084)	<.001
UACR	ref	1.169(0.671-2.035)	.581	1.334(0.800-2.223)	0.269	2.076(1.163-3.707)	.013
UHR	ref	1.096(0.631-1.906)	.745	1.436(0.739-2.789)	0.286	2.922(1.623-5.259)	<.001
UAR	ref	1.521(0.869-2.662)	.142	1.621(0.939-2.797)	0.083	2.252(1.311-3.869)	.003

BUN/Cr, Blood urea nitrogen-to-creatinine ratio; MASLD, Metabolic dysfunction–associated steatotic liver disease; SUA/Cr, Serum uric acid-to-creatinine ratio; UACR, Urinary albumin-to-creatinine ratio; UAR, Uric acid-to-albumin ratio; UHR, Uric acid-to-high-density lipoprotein cholesterol ratio.

Supplementary Table 8. Adjusted logistic regression for significant fibrosis with urinary metabolic indicators after PSM (Model 3).

		Q2		Q3		Q4	
Index	Q1	OR (95%CI)	*P* value	OR (95%CI)	*P* value	OR (95%CI)	*P* value
SUA/Cr	ref	1.075(0.493-2.344)	.856	1.555(0.716-3.380)	0.265	1.171(1.021-1.343)	.024
UHR	ref	1.139(0.643-2.017)	.655	1.410(0.626-3.175)	0.407	2.067(1.037-4.118)	.039
UAR	ref	1.455(0.601-3.523)	.406	1.802(0.803-4.045)	0.154	3.917(1.701-9.018)	.001

SUA/Cr, Serum uric acid-to-creatinine ratio; UACR, Urinary albumin-to-creatinine ratio; UAR, Uric acid-to-albumin ratio; UHR, Uric acid-to-high-density lipoprotein cholesterol ratio.
